# Mechanisms Associated with PINK1 Mutations in Parkinson's Disease

**DOI:** 10.12688/f1000research.170090.1

**Published:** 2025-10-20

**Authors:** Hanliang Dan, Xiaohui Huang, Zheng Liu, Bing Wei, Maslinda Musa

**Affiliations:** 1 School of Biology, Faculty of Applied Sciences, Universiti Teknologi MARA, UiTM, Shah Alam Seksyen 2, 40450 Selangor, Malaysia; 2 Centre for Chemical Synthesis and Polymer Technology, Institute of Science, Universiti Teknologi MARA, UiTM, Shah Alam Seksyen 2, 40450 Selangor, Malaysia; 3 Nanxishan Hospital of Guangxi Zhuang Autonomous Region (The Second People's Hospital of Guangxi Zhuang Autonomous Region), Guilin, Guangxi 541002, China; 4 Guangxi Key Laboratory of Multimodal Biomarkers and Precision Diagnosis, College of Medical Laboratory and Biotechnology, Guilin Medical University, Guilin, Guangxi, 541004, China; 5 Guilin Medical University, Guangxi Key Laboratory of Tumor Immunology and Microenvironment Regulation, Department of Basic Medicine, Guilin, Guangxi 541004, China

**Keywords:** Parkinson's disease, PINK1, genetic mutation, mitochondrial phosphorylation, autophagy pathways, oxidative stress

## Abstract

Parkinson’s disease (PD) is a widespread and progressively debilitating neurodegenerative disorder, with its global prevalence steadily increasing. Central to its pathogenesis is the PINK1 gene (PTEN-induced putative kinase 1), which plays a pivotal role in mitochondrial function and protecting cells from oxidative stress-induced apoptosis. This review explores the complex relationship between PINK1 mutations and PD, highlighting their involvement in key pathogenic mechanisms. It discusses how these mutations contribute to mitochondrial dysfunction, alterations in phosphorylation pathways, impaired autophagy, and heightened sensitivity to oxidative stress. These insights lay the groundwork for future research and therapeutic approaches, aiming to address the urgent need for effective PD interventions.

## 1. Introduction

Parkinson’s disease (PD) is a common, chronic, and progressive neurodegenerative disorder, second only to Alzheimer’s disease in prevalence. It affects both the central nervous system and peripheral organs, representing a growing health challenge as the global population ages. According to the *World Health Organization’s Parkinson’s Disease Report*, more than 8.5 million people were living with PD in 2019, an 81% increase since 2000. During the same period, PD-related deaths doubled to 329,000. A meta-analysis of four North American populations revealed a strong age-related trend in prevalence: less than 1% among men and women aged 45–54 years, increasing to 4% in men and 2% in women aged 85 years and older. ^1^

The hallmark motor symptoms of PD include tremors, muscle rigidity, bradykinesia, postural instability, and reduced facial expression. The etiology of PD is multifactorial, likely involving interactions between genetic predisposition and environmental exposures. Established environmental risk factors include rural living, agricultural work, well-water consumption, and pesticide exposure. ^2^ Pathologically, PD is characterized by the degeneration of dopaminergic neurons in the substantia nigra. Within four years of diagnosis, there is a rapid and nearly complete loss of dopaminergic markers in the dorsal striatum, while the loss of melanin-containing neurons in the substantia nigra occurs with a relative delay. ^3^

PINK1 (PTEN-induced putative kinase 1) mutations represent the second most common cause of autosomal recessive early-onset Parkinson’s disease (EOPD), following *Parkin* mutations. ^4^ The PINK1 gene encodes a 581–amino acid protein that is broadly expressed and structurally composed of an N-terminal mitochondrial targeting sequence, a transmembrane domain, and a C-terminal serine/threonine kinase domain. ^5^ Functionally, PINK1 plays a critical role in protecting cells from oxidative stress–induced apoptosis. Increasing research efforts are directed toward clarifying the biological functions of this serine/threonine kinase, with the goal of informing therapeutic development for PD. ^6^ The objective of this review is to examine the relationship between PINK1 mutations and Parkinson’s disease, and to elucidate the complex mechanisms involved, thereby providing valuable insights into potential therapeutic strategies.

## 2. The diversity of PINK1 mutations

Mutations in the PINK1 gene are strongly linked to PD. As a mitochondria-associated kinase, PINK1 plays a pivotal role in regulating mitochondrial biogenesis, quality control, and clearance. Loss or dysfunction of PINK1 disrupts these processes, leading to mitochondrial abnormalities characterized by altered morphology, reduced mitochondrial membrane potential (Δψm), and elevated reactive oxygen species (ROS) production, thereby promoting neurodegeneration.

Most pathogenic mutations in PINK1 occur within its kinase domain and include nonsense, missense, and deletion variants. ^7^ In 2004, Valente et al. first identified PINK1 mutations in three consanguineous families of the PARK6 pedigree, reporting two homozygous mutations within the kinase domain: a missense mutation (G309D) and a truncation mutation (W437X). ^8^ That same year, Hatano et al. identified six novel pathogenic variants (R246X, H271Q, E417G, L347P, and Q239X/R492X) in six unrelated PARK6 families. ^9^ Subsequent studies further revealed heterozygous mutations in sporadic early-onset PD, highlighting the role of PINK1 in both familial and sporadic disease forms. ^10^ Expanding research across diverse populations worldwide has identified additional homozygous and heterozygous mutations, many of which are predicted to impair or abolish kinase activity. *In vitro* analyses confirm that mutations such as G309D, L437P, G386A, and G409V markedly reduce kinase function. ^7, 11^

Collectively, these studies provide comprehensive evidence of the close association between PINK1 mutations and PD, underscoring its critical role in early-onset familial and sporadic disease. A deeper understanding of the structural and functional consequences of these mutations has advanced our knowledge of mitochondrial regulation in PD pathogenesis and offers valuable guidance for the development of future therapeutic interventions.

## 3. PINK1 and the pathogenesis of PD

### 3.1 PINK1 mutations and mitochondrial phosphorylation

Investigating the relationship between PINK1 mutations and phosphorylation pathways offers important insights into the molecular mechanisms underlying PD. Pridgeon et al. demonstrated that PINK1 kinase protects cells against oxidative stress–induced apoptosis by phosphorylating the mitochondrial chaperone TRAP1. However, PD-associated PINK1 mutations (G309D, L347P, and W437X) disrupt this protective mechanism, implicating them in disease pathogenesis. ^12^

Similarly, Plun-Favreau and colleagues identified PINK1’s interaction with the mitochondrial serine protease HtrA2/OMI. ^13^ Under physiological conditions, PINK1 activates the p38 pathway, which mediates HtrA2 phosphorylation and enhances its protease activity, thereby conferring resistance to cellular stress. In PD patients carrying PINK1 mutations such as C575R or Y431H, impaired HtrA2 phosphorylation diminishes this protective response, heightening vulnerability to mitochondrial stress. ^13^ Supporting evidence from Drosophila models confirmed that HtrA2/OMI functions downstream of PINK1 in a shared pathway, although independent of Parkin. ^14, 15^

Beyond HtrA2, Parkin itself has been identified as a direct substrate of PINK1. Di Sha et al. showed that PINK1 phosphorylates Parkin, thereby activating its E3 ligase activity, promoting K63-linked polyubiquitination, and enhancing NF-κB–mediated ubiquitin signaling. Notably, pathogenic PINK1 mutations (G309D and L347P) or kinase-inactive PINK1 abrogate this effect, abolishing Parkin phosphorylation and disrupting downstream signaling. ^16^

Further insights from Drosophila models highlight the essential role of PINK1 autophosphorylation at serine 346 in regulating the PINK1/Parkin pathway. Loss of this phosphorylation event impairs Parkin mitochondrial translocation and activation, ultimately exacerbating photoreceptor neurodegeneration. These findings emphasize the critical role of PINK1 autophosphorylation in mitochondrial quality control and neuronal survival. ^17^

**Figure 1.**
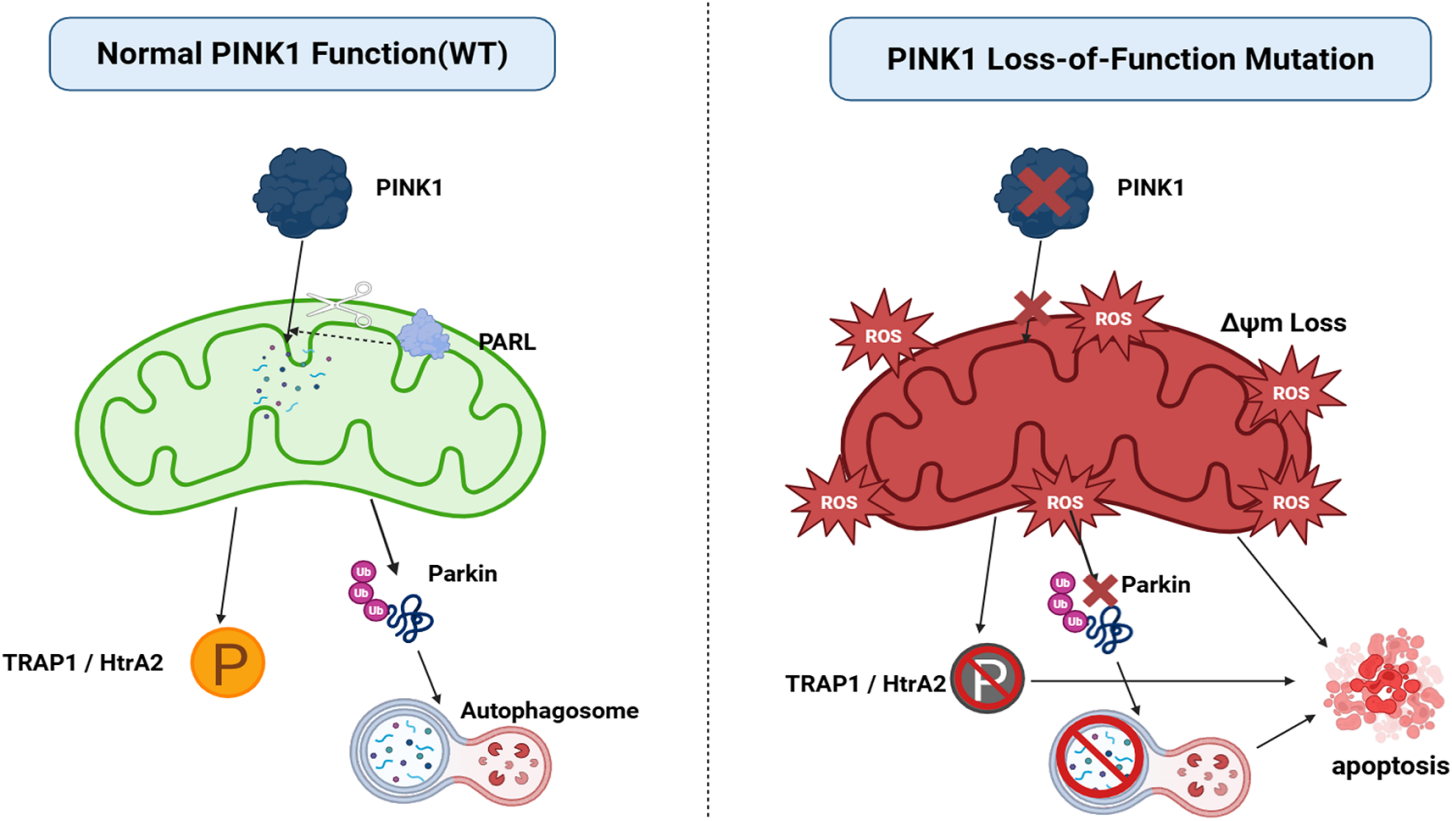
Proposed mechanisms by which PINK1 dysfunction contributes to the pathogenesis of Parkinson’s disease.

Collectively, these studies demonstrate how PINK1 mutations compromise key phosphorylation pathways, thereby promoting mitochondrial dysfunction and neurodegeneration ( Figure 1). A deeper understanding of these processes not only clarifies PD pathogenesis but also highlights potential targets for therapeutic intervention.

### 3.2 PINK1 mutations and abnormal autophagy pathways

Mitophagy, the selective removal of damaged mitochondria via autophagosomes, is essential for maintaining mitochondrial quality and regulating mitochondrial number under varying cellular conditions.

In Parkinson’s disease, Parkin, an E3 ubiquitin ligase, plays a central role in mitochondrial quality control. ^18^ Loss of PINK1 function induces mitochondrial fragmentation and defective autophagy, a phenotype that can be rescued by PINK1 re-expression. ^19^ Under normal conditions, PINK1 undergoes voltage-dependent degradation by the mitochondrial inner membrane rhomboid protease PARL. ^20, 21^ However, when mitochondrial membrane potential collapses, PINK1 accumulates on the outer mitochondrial membrane of damaged mitochondria, serving as a signal for their clearance. This accumulated PINK1 recruits Parkin and activates it by phosphorylating ubiquitin at serine 65, establishing ubiquitin as a bona fide PINK1 substrate. ^22^ Phosphorylated ubiquitin allosterically promotes the release of UBCH7 ubiquitin, relieving Parkin’s autoinhibition and enhancing its E3 ligase activity. ^18, 23^ Once translocated to the mitochondrial surface, Parkin ubiquitinates multiple outer mitochondrial membrane (OMM) proteins, facilitating the recruitment of additional factors that initiate mitophagy. ^24^ In parallel, the ubiquitin-binding adaptor p62 (sequestosome 1) contributes by clustering ubiquitylated proteins through polymerization and recruiting them into autophagosomes via LC3 binding, thereby supporting constitutive basal autophagy. ^25^

Pathogenic PINK1 mutations disrupt this process by impairing both kinase activity and interaction with Parkin, thereby hindering the selective clearance of depolarized mitochondria and contributing to PD pathogenesis ( Figure 1). Geisler et al. demonstrated that PINK1 mutants W437X, Q126P, G309D, and L347P prevent Parkin translocation and block clearance of damaged mitochondria during mitophagy. ^26^ Interestingly, other studies suggest that these same variants, while impairing Parkin recruitment and kinase activity, may partially correct the aberrant autophagy induced by complete PINK1 deletion. ^27^

### 3.3 PINK1 mutations and oxidative stress

In 1983, Langston and colleagues reported that several individuals developed Parkinsonian symptoms following intravenous exposure to a banned compound, later identified as 1-methyl-4-phenyl-1,2,5,6-tetrahydropyridine (MPTP), suggesting its potential role as a PD trigger. ^28^ Structurally analogous to meperidine, MPTP itself is not directly toxic. After crossing the blood–brain barrier (BBB), it is metabolized by astrocytic enzymes into 1-methyl-4-phenyl-2,3-dihydropyridine, which is subsequently oxidized to the toxic metabolite 1-methyl-4-phenylpyridinium (MPP ^+^) within the central nervous system. ^29, 30^

Under normal conditions, electrons pass through mitochondrial respiratory chain complexes (I–IV) to generate ATP, with reactive oxygen species (ROS) produced as byproducts of oxygen metabolism. ^31^ While ROS are important for immune defense and cell signaling, their overproduction or impaired clearance leads to oxidative stress, which damages DNA, proteins, and lipids. ^32^ MPP ^+^ inhibits mitochondrial NADH dehydrogenase (complex I), resulting in excessive ROS generation and subsequent degeneration of dopaminergic neurons in the nigrostriatal pathway. ^33^

PINK1 plays a critical protective role in neuronal mitochondria ( Figure 1). Human neurons deficient in PINK1 display profound oxidative stress, widespread mitochondrial dysfunction, and abnormal morphology. ^34^ Deng et al. demonstrated that reduced PINK1 expression via siRNA decreased cell viability and increased susceptibility to MPP ^+^- and rotenone-induced cytotoxicity in SH-SY5Y cells, reinforcing PINK1’s role in dopaminergic neuron survival. ^35^ Further, Tang et al. identified novel heterozygous missense mutations—DJ-1 A39S and PINK1 P399L—in a Chinese family with early-onset PD. Their findings showed that DJ-1 and PINK1 form a protective complex against oxidative stress, while the disease-associated DJ-1 A39S and PINK1 P399L variants disrupt this interaction, thereby increasing vulnerability to neurotoxin-induced cell death. ^36^

## 4. Conclusions

In summary, this review highlights the intricate relationship between PINK1 mutations and Parkinson’s disease, emphasizing their effects on mitochondrial function, phosphorylation pathways, autophagy, and oxidative stress. By disrupting mitochondrial quality control, these mutations contribute to the cascade of events leading to neurodegeneration in PD. A deeper understanding of these pathogenic mechanisms not only provides a more comprehensive perspective on disease progression but also lays the groundwork for the development of targeted therapeutic strategies to address the challenges posed by Parkinson’s disease.

## Data Availability

No data are associated with this article.
