# Mechanisms Associated with PINK1 Variants in Parkinson's Disease

**DOI:** 10.12688/f1000research.170090.2

**Published:** 2026-04-22

**Authors:** Hanliang Dan, Xiaohui Huang, Zheng Liu, Bing Wei, Maslinda Musa

**Affiliations:** 1 School of Biology, Faculty of Applied Sciences, Universiti Teknologi MARA, UiTM, Shah Alam Seksyen 2, 40450 Selangor, Malaysia; 2 Centre for Chemical Synthesis and Polymer Technology, Institute of Science, Universiti Teknologi MARA, UiTM, Shah Alam Seksyen 2, 40450 Selangor, Malaysia; 3 Nanxishan Hospital of Guangxi Zhuang Autonomous Region (The Second People's Hospital of Guangxi Zhuang Autonomous Region), Guilin, Guangxi 541002, China; 4 Guangxi Key Laboratory of Multimodal Biomarkers and Precision Diagnosis, College of Medical Laboratory and Biotechnology, Guilin Medical University, Guilin, Guangxi, 541004, China; 5 Guilin Medical University, Guangxi Key Laboratory of Tumor Immunology and Microenvironment Regulation, Department of Basic Medicine, Guilin, Guangxi 541004, China

**Keywords:** Parkinson's disease, PINK1, genetic mutation, mitochondrial phosphorylation, autophagy pathways, oxidative stress

## Abstract

Parkinson’s disease (PD) is a widespread and progressively debilitating neurodegenerative disorder with a growing global prevalence. While most cases are sporadic, loss-of-function variants in the *PINK1* gene are a primary cause of autosomal recessive early-onset PD. This review critically explores the molecular mechanisms linking PINK1 dysfunction to PD, with a specific focus on the kinase’s role in phosphorylating Ubiquitin and Parkin at the conserved Serine 65 (Ser65) residue. We discuss how this phosphorylation event acts as a molecular switch to recruit the novel autophagy receptors Optineurin (OPTN) and NDP52, thereby initiating mitophagy—a process often disrupted by pathogenic variants. Furthermore, we examine the emerging role of PINK1 in suppressing neuroinflammation via the STING pathway and evaluate the translational potential of targeting these molecular checkpoints for therapeutic intervention. These insights lay the groundwork for developing precision medicine strategies to address the urgent need for effective PD treatments.

## 1. Introduction

Parkinson’s disease (PD) is the second most prevalent neurodegenerative disorder worldwide, characterized by the progressive loss of dopaminergic neurons in the substantia nigra pars compacta. According to recent epidemiological data, the global prevalence of PD has more than doubled over the past two decades, a trend largely attributed to aging populations. ^1^ ^,^ ^2^ While the majority of cases are idiopathic, approximately 5–10% of patients exhibit monogenic inheritance. Among these, loss-of-function variants in the *PINK1* gene (PTEN-induced kinase 1; HGNC:14581) are established as a primary cause of autosomal recessive early-onset PD (EOPD). Although *PINK1*-related PD represents a subset of the total patient population, elucidating its role is critical for understanding the mitochondrial quality control failure that characterizes both familial and sporadic forms of the disease. ^3^

The clinical hallmark of PD is the progressive degeneration of dopaminergic neurons within the substantia nigra pars compacta, leading to classic motor symptoms such as bradykinesia, resting tremor, and rigidity. ^4^ However, it is increasingly recognized that a prolonged prodromal phase, characterized by non-motor symptoms including hyposmia, sleep disturbances, and depression, often precedes motor onset by decades. ^5^ In the context of PINK1-linked PD, the clinical phenotype typically manifests as early-onset Parkinsonism, frequently occurring before the age of 45. ^3^ Notably, patients with *PINK1* variants often exhibit a slower disease progression and a sustained, robust response to levodopa compared to those with late-onset sporadic PD. ^6^ Understanding these distinct clinical trajectories is crucial for accurate prognosis and personalized patient management.

The *PINK1* gene encodes a 581-amino acid protein that is broadly expressed and structurally composed of an N-terminal mitochondrial targeting sequence, a transmembrane domain, and a C-terminal serine/threonine kinase domain. ^7^ While loss-of-function variants in *PINK1* are widely recognized for impairing mitochondrial protection against oxidative stress, ^8^ the precise molecular cascade linking these deficits to clinical neurodegeneration remains incompletely mapped. Specifically, there is a critical knowledge gap regarding how PINK1 dysfunction extends beyond canonical mitophagy to affect broader cellular processes such as innate immunity and proteostasis. ^9^ Moreover, despite extensive mechanistic studies, translating these findings into effective therapeutics has proven difficult. Therefore, the objective of this review is to critically examine the multifaceted mechanisms of PINK1 *-*driven pathogenesis—moving beyond classical mitophagy—and to evaluate the translational potential of targeting this pathway for PD intervention.

## 2. The diversity of PINK1 mutations

Under basal physiological conditions, the cellular levels of PINK1 are maintained at an exceptionally low limit through a rapid and constitutive turnover mechanism. The PINK1 precursor is imported into healthy, polarized mitochondria via the translocase of the outer membrane (TOM) and translocase of the inner membrane (TIM) complexes. ^10^ Upon reaching the inner mitochondrial membrane (IMM), the N-terminal mitochondrial targeting sequence (MTS) is first cleaved by the mitochondrial processing peptidase (MPP). Subsequently, the transmembrane domain is cleaved by the rhomboid protease PARL (presenilin-associated rhomboid-like) between Alanine-103 and Phenylalanine-104. ^11^ This proteolytic processing generates a truncated, unstable form of PINK1, which is retro-translocated to the cytosol and rapidly degraded by the ubiquitin-proteasome system via the N-end rule pathway. ^12^ However, this homeostatic cycle is intimately coupled to mitochondrial bioenergetics. When mitochondria sustain damage and lose their membrane potential (Δψm), the TIM23-mediated import of PINK1 is arrested. Consequently, full-length PINK1 stabilizes on the outer mitochondrial membrane (OMM) with its kinase domain facing the cytosol. ^13^ This accumulation promotes PINK1 homodimerization and trans-autophosphorylation at Serine 228 and Serine 402, events that are requisite for maximizing its kinase activity and initiating the downstream recruitment of Parkin. ^14^ Thus, PINK1 functions as a molecular sensor of mitochondrial quality, converting bioenergetic stress into a distinct phosphorylation signal.

## 3. The diversity of PINK1 pathogenic variants

The integrity of the mitochondrial quality control system is fundamentally dependent on the precise catalytic activity of PINK1. Consequently, pathogenic variants in the *PINK1* gene predominantly result in a loss of function, disrupting the neuroprotective response described in the previous section. Since the initial identification of the G309D missense variant and the W437X truncation in the PARK6 pedigree by Valente et al., ^15^ over 70 distinct pathogenic variants have been cataloged. Structurally, these variants are not randomly distributed but are heavily clustered within the highly conserved serine/threonine kinase domain (residues 156–509), underscoring the critical importance of kinase activity for neuroprotection. ^16^

Mechanistically, these variants impair PINK1 function through distinct molecular deficits. A significant proportion of missense variants, such as G309D, L347P, and G409V, induce kinase inactivation by destabilizing the ATP-binding pocket or the catalytic loop. This structural compromise abolishes the essential autophosphorylation events (e.g., at Ser228 and Ser402) required for the recruitment of Parkin. ^17^ Beyond catalytic inactivation, protein instability represents another major pathogenic mechanism. Truncation variants, including W437X and Q456X, often result in the rapid degradation of the transcript via the nonsense-mediated decay pathway or produce unstable protein fragments that fail to accumulate on the outer mitochondrial membrane, even under conditions of cellular stress. ^18^ Furthermore, rare variants located in the N-terminal mitochondrial targeting sequence have been observed to interfere with the efficient import of PINK1 into mitochondria, thereby preventing its correct subcellular localization. ^19^

Collectively, regardless of whether the defect is kinetic (kinase dead) or structural (instability), these perturbations converge on a common pathological outcome: the failure to sense mitochondrial depolarization. This sensory defect prevents the initiation of mitophagy, leading to the progressive accumulation of dysfunctional organelles within dopaminergic neurons.

**Figure 1.**
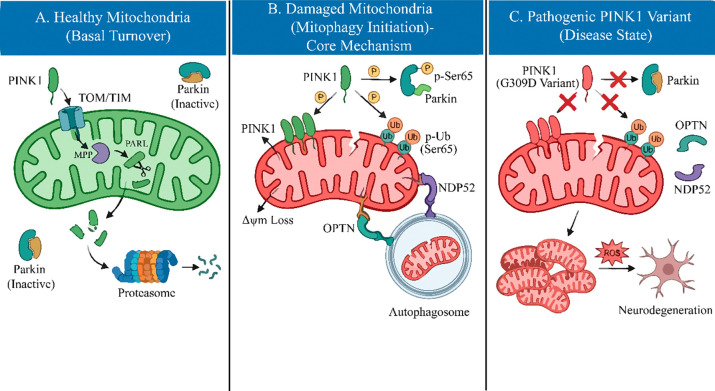
(A) Healthy Mitochondria (Basal Turnover): Under physiological conditions, PINK1 is constitutively imported into the inner mitochondrial membrane via the TOM/TIM complex. It is processed by the mitochondrial processing peptidase (MPP) and the rhomboid protease PARL, generating cleaved fragments that are rapidly degraded by the proteasome. This continuous turnover maintains low levels of PINK1, keeping Parkin inactive in the cytosol. (B) Damaged Mitochondria (Mitophagy Initiation): Upon mitochondrial damage (loss of membrane potential (Δψm), PINK1 import is arrested, leading to the accumulation of full-length PINK1 on the outer mitochondrial membrane (OMM). Stabilized PINK1 phosphorylates both Parkin and Ubiquitin (Ub) at the conserved Serine 65 (Ser65) residue. Phosphorylated Ubiquitin (p-Ub) serves as a signal to recruit the primary autophagy receptors Optineurin (OPTN) and NDP52. These receptors bridge the ubiquitinated mitochondria to the autophagosome via LC3, initiating mitophagy. (C) Pathogenic PINK1 Variant (Disease State): Pathogenic variants (e.g., G309D) located in the kinase domain impair the catalytic activity of PINK1. Consequently, PINK1 fails to phosphorylate Ubiquitin and Parkin even under stress conditions. The absence of p-Ub prevents the recruitment of OPTN and NDP52, blocking the formation of the autophagosome. This failure in quality control leads to the accumulation of damaged, ROS-producing mitochondria, ultimately driving dopaminergic neurodegeneration.

## 4. PINK1 and the pathogenesis of PD

### 4.1 PINK1 variants impair phosphorylation of downstream substrates

Investigating the interplay between PINK1 kinase activity and its substrates provides crucial insights into PD pathogenesis. Contrary to earlier assumptions that PINK1 broadly phosphorylates mitochondria, it targets specific proteins to mediate neuroprotection. Early studies identified the mitochondrial chaperone TRAP1 and the serine protease HtrA2/OMI as potential substrates. Pridgeon et al. demonstrated that PINK1 phosphorylates TRAP1 to suppress oxidative stress, a function abolished by the G309D variant. ^8^ Similarly, PINK1-dependent phosphorylation of HtrA2 enhances its protease activity, conferring resistance to cellular stress. ^20^

However, the most significant breakthrough has been the identification of Parkin and Ubiquitin as the physiological substrates of PINK1. Under stress, PINK1 phosphorylates both the ubiquitin-like (UBL) domain of Parkin and Ubiquitin itself at the conserved Serine 65 (Ser65) residue. ^21^ ^,^ ^22^ This dual phosphorylation event is the molecular switch that activates Parkin’s E3 ligase activity ( Figure 1). Crucially, pathogenic variants such as G309D and L347P fail to phosphorylate Ubiquitin or Parkin at Ser65, thereby locking Parkin in an auto-inhibited state and completely blocking the downstream quality control cascade. ^23^

### 4.2 Disruption of mitophagy: The role of OPTN and NDP52

Mitophagy, the selective degradation of damaged mitochondria, is the central pathway regulated by PINK1. In the absence of functional PINK1, this clearance mechanism fails. While early models suggested that p62/SQSTM1 was the primary autophagy receptor linking ubiquitinated mitochondria to the autophagosome, recent evidence has redefined this model.

Current consensus indicates that Optineurin (OPTN) and NDP52 (CALCOCO2) are the primary autophagy receptors recruited by *PINK1/Parkin*-mediated ubiquitin chains. ^24^ These receptors bind to the ubiquitinated outer mitochondrial membrane and recruit the autophagy machinery via their LC3-interacting regions. Importantly, PINK1 further enhances this process by phosphorylating ubiquitin chains, which serves as a “eat-me” signal that selectively recruits OPTN and NDP52. ^25^ Pathogenic *PINK1* variants disrupt this recruitment hierarchy. Without the initial phosphorylation trigger from PINK1, Parkin is not activated, ubiquitin chains are not formed, and OPTN/NDP52 cannot engage the autophagy machinery. Consequently, damaged mitochondria accumulate, releasing toxic byproducts that drive dopaminergic neurodegeneration.

### 4.3 Beyond mitophagy: PINK1 and neuroinflammation

Emerging evidence suggests that the consequences of PINK1 dysfunction extend beyond defective mitophagy to include aberrant innate immune signaling. Under physiological conditions, mitophagy prevents the leakage of mitochondrial DNA (mtDNA) into the cytosol. However, in the absence of PINK1, damaged mitochondria accumulate and release mtDNA, which is sensed by the cGAS-STING pathway. This triggers a robust Type I interferon response and the release of pro-inflammatory cytokines such as Interleukin-6 (IL-6). ^26^ Crucially, Matheoud et al. demonstrated that intestinal infection with Gram-negative bacteria in *Pink1*-knockout mice engages mitochondrial antigen presentation and autoimmune mechanisms, highlighting the gut-brain axis and inflammation as key drivers of pathogenesis. ^27^ Clinically, elevated serum IL-6 levels have been reported in patients with PINK1 or *PRKN* variants, correlating with disease progression. ^28^ This suggests that PINK1 functions not only as a quality control sensor but also as a critical immunological checkpoint.

### 4.4 Therapeutic implications

Given that *PINK1* loss-of-function drives PD, pharmacological strategies to amplify PINK1 activity or bypass its function are being actively explored. One approach involves the neo-substrate Kinetin (N6-furfuryladenine) and its riboside prodrugs, which have been shown to accelerate PINK1 activation and enhance Parkin recruitment in neuronal cells independent of mitochondrial depolarization. ^29^ ^,^ ^30^ Additionally, inhibitors of the deubiquitinase USP30, which opposes PINK1/Parkin signaling by removing ubiquitin chains from mitochondria, have shown promise in preclinical models by restoring mitophagy flux. ^31^ Recent studies highlight that USP30 inhibition can rescue mitophagy defects even in the presence of certain pathogenic variants, making it a viable therapeutic target. ^32^ These targeted approaches represent the frontier of precision medicine for *PINK1*-linked Parkinson’s disease.

## 5. Conclusions

In summary, this review highlights the intricate relationship between *PINK1* loss-of-function variants and the pathogenesis of Parkinson’s disease. The recent identification of Optineurin (OPTN) and NDP52 as the primary autophagy receptors recruited by PINK1-phosphorylated ubiquitin chains has redefined our understanding of mitophagy. Pathogenic variants, by failing to execute the critical Ser65 phosphorylation step, block this recruitment hierarchy, leading to the accumulation of damaged mitochondria. Beyond bioenergetic failure, recent evidence underscores that this defect triggers the cGAS-STING pathway, driving neuroinflammation as a key component of disease progression. A deeper understanding of these mechanisms identifies specific therapeutic targets—such as amplifying PINK1 kinase activity with Kinetin or inhibiting the deubiquitinase USP30—offering promising new avenues for clinical intervention in Parkinson’s disease.

## Data Availability

No data are associated with this article.
