# Supplementary material for: Mechanisms Associated with PINK1 Variants in Parkinson's Disease
Source: F1000Res. 2026 Jun 18;14:1138. Originally published 2025 Oct 20. [Version 3] doi: 10.12688/f1000research.170090.3 (PMC13294661; doi:10.12688/f1000research.170090.3)
Supplement: Peer review history

# Reviewer report: Reviewer response for version 3

**DOI:** 10.5256/f1000research.202783.r494992

**Published:** 2026-07-13

**Authors:** Ryan L Davis

**Affiliations:** 1 University of Sydney, Sydney, Sydney, Australia

The manuscript is vastly improved from the first version. I'm still not convinced it makes a considerable conceptual contribution to advancing the perspective or understanding of the topic and .

 There are a few things i noticed that should be addressed.

 1. In the introduction it is mentioned about cardianl clinical signs and then the prodrome. No mention of brain-first and body-first, which would link with the mention of the gut later on. More could be made of the microbiome link to PD initiation, given the mention of intestinal infection induced PD in PINK1 mice later on. 

 2. the current section 2 title does not match the content. Likely a hangover from an earlier version as section 3 has a similar title. Requires updating. 

 3. the content in section 2 should be distilled considerably to include only the necessary information (are specific amino acids or the import and cleavage process detail necessary and relevant to the review and the information presented after??). The word count saved could be used to discuss a different non-mitophagy impact of PINK1 dysfunction, or add some detail to the intervention section (4.4) as this was a key feature of the the review objective outlined at the end of the introduction.

 4. In the second sentence of the first paragraph of section 3 it mentions "... neuroprotective response described in the previous section". But the previous section doesn't discuss neuroprotection.

 5. Is it worth mentioning glial cells and their role in neurodegeneration through impaired neuroprotection? What about mitochondrial transfer vis nanotubes between neurons and glial cells? Is it known whether oxidative stress from impaired mitophagy is alone sufficient to "drive" neurodegeneration or is it part of a broader spectrum of damage and dysfunction? Nitrosative stress? mitochondrial UPR?

 6. For the therapeutic section, what about attempts to increase mitochondrial biogenesis, or over-express Parkin, or use high strength bioavailable antioxidants to counteract the oxidative stress? What is the state of clinical trials? Even if there aren't any, that is an important point to make .

 The manuscript has improved but still lacks clarity, comprehension and execution of intent. It is not my intention to further aid you in re-writing the manuscript via my critique and suggestions . 

 If the reviewer consensus is to accept and the editor is happy with the article, i wish you well . 

Is the review written in accessible language?

Yes

Are all factual statements correct and adequately supported by citations?

Partly

Are the conclusions drawn appropriate in the context of the current research literature?

No

Is the topic of the review discussed comprehensively in the context of the current literature?

No

Reviewer Expertise:

Parkinson's disease, genomics, mitochondrial disease, molecular pathology

I confirm that I have read this submission and believe that I have an appropriate level of expertise to state that I do not consider it to be of an acceptable scientific standard, for reasons outlined above.

# Reviewer report: Reviewer response for version 3

**DOI:** 10.5256/f1000research.202783.r494991

**Published:** 2026-06-24

**Authors:** DOMENICO PRATICO

**Affiliations:** 1 Department of Neural Sciences, Lewis Katz School of Medicine at Temple University, Philadelphia, Pennsylvania, USA

The authors have addressed all my comments in a satisfactory fashion.

Is the review written in accessible language?

Yes

Are all factual statements correct and adequately supported by citations?

Yes

Are the conclusions drawn appropriate in the context of the current research literature?

Yes

Is the topic of the review discussed comprehensively in the context of the current literature?

Yes

Reviewer Expertise:

Brain health; Brain aging; Neurodegeneration

I confirm that I have read this submission and believe that I have an appropriate level of expertise to confirm that it is of an acceptable scientific standard.

# Reviewer report: Reviewer response for version 2

**DOI:** 10.5256/f1000research.197781.r481886

**Published:** 2026-05-25

**Authors:** DOMENICO PRATICO

**Affiliations:** 1 Department of Neural Sciences, Lewis Katz School of Medicine at Temple University, Philadelphia, Pennsylvania, USA

The authors have addressed most of the comments that were raised by the previous reviewers. The paper is improved.

 However, some minor issues still remain.

 1. Introduction.

 First paragraph. Say which is the most prevalent neurodegenerative disorder.

 Second paragraph. Delete "the progressive degeneration of dopaminergic neurons within the substantia nigra pars compacta". Here it is only the clinical part of the disease that should be described.

 Third paragraph. Proteostasis is mentioned, but this concept is never developed.

 2. The diversity of PINK1 mutations.

 In this paragraph you should mention Figure 1, which is never mentioned throughout the paper.

 Section 4.3. 

 The authors should connect better neuroinflammation with PD pathogenesis.

 Is the disruption of mitophagy that triggers neuroinflammation? or neuroinflammation is independent from mitophagy?

 5. Conclusion. Last sentence. Add "can help to identify" after "A deeper understanding of these mechanisms.....

 Spell out the acronyms such as TIM23, TRAP1, etc. etc. 

 Use PD instead of Parkinson's disease consistently throughout the text (as an example, see Conclusion). 

Is the review written in accessible language?

Yes

Are all factual statements correct and adequately supported by citations?

Yes

Are the conclusions drawn appropriate in the context of the current research literature?

Yes

Is the topic of the review discussed comprehensively in the context of the current literature?

Yes

Reviewer Expertise:

Brain health; Brain aging; Neurodegeneration

I confirm that I have read this submission and believe that I have an appropriate level of expertise to confirm that it is of an acceptable scientific standard, however I have significant reservations, as outlined above.

# Response

**Published:** 2026-05-29

**Authors:** Zheng Liu

**Affiliations:** Guilin Medical University, China

The authors have addressed most of the comments that were raised by the previous reviewers. The paper is improved.

 However, some minor issues still remain.

 1. Introduction.

 First paragraph. Say which is the most prevalent neurodegenerative disorder.

 Second paragraph. Delete "the progressive degeneration of dopaminergic neurons within the substantia nigra pars compacta". Here it is only the clinical part of the disease that should be described.

**Response:**

 We thank the reviewer for these helpful suggestions regarding the Introduction.

 In the first paragraph, we have revised the opening sentence to clarify that Alzheimer's disease (AD) is the most prevalent neurodegenerative disorder worldwide, followed by Parkinson's disease (PD) as the second most common condition.

 In the second paragraph, we have removed the pathological description "the progressive degeneration of dopaminergic neurons within the substantia nigra pars compacta" and now focus exclusively on the clinical presentation: "The clinical hallmark of PD comprises classic motor symptoms such as bradykinesia, resting tremor, and rigidity."

 Third paragraph. Proteostasis is mentioned, but this concept is never developed.

**Response:**

 We thank the reviewer for this observation. We have removed the term "proteostasis" from the third paragraph of the Introduction, as this concept was not developed elsewhere in the manuscript.

 2. The diversity of PINK1 mutations.

 In this paragraph you should mention Figure 1, which is never mentioned throughout the paper.

**Response:**

 We have revised Section 2 to include a reference to Figure 1, which illustrates how pathogenic PINK1 variants disrupt the mitochondrial quality control pathway. The figure is now explicitly cited to support the discussion of loss-of-function mechanisms.

 Section 4.3. 

 The authors should connect better neuroinflammation with PD pathogenesis.

 Is the disruption of mitophagy that triggers neuroinflammation? or neuroinflammation is independent from mitophagy?

**Response:**

 We thank the reviewer for this important question. We have added an explicit statement in Section 4.3 clarifying that neuroinflammation is not independent from mitophagy; instead, mitophagy disruption directly triggers neuroinflammation. The subsequent discussion on the cGAS-STING pathway, gut-brain axis evidence, and clinical IL-6 data in PINK1/PRKN patients has been retained, as these findings directly connect neuroinflammation to PD pathogenesis.

 5. Conclusion. Last sentence. Add "can help to identify" after "A deeper understanding of these mechanisms.....

 Spell out the acronyms such as TIM23, TRAP1, etc. etc. 

 Use PD instead of Parkinson's disease consistently throughout the text (as an example, see Conclusion). 

**Response:**

 We thank the reviewer for these helpful suggestions.

 We have revised the final sentence of the Conclusion to add "can help to identify" as requested. All acronyms, including TIM23 (translocase of the inner membrane 23), TRAP1 (TNF receptor-associated protein 1), UBL (ubiquitin-like), LC3 (microtubule-associated protein 1 light chain 3), mtDNA (mitochondrial DNA), cGAS (cyclic GMP-AMP synthase), STING (stimulator of interferon genes), and USP30 (ubiquitin-specific protease 30), are now spelled out at their first appearance. Additionally, we have replaced all instances of "Parkinson's disease" with "PD" throughout the text, except for the first appearance in the Abstract where the full term is introduced.

# Reviewer report: Reviewer response for version 1

**DOI:** 10.5256/f1000research.187509.r425648

**Published:** 2025-12-30

**Authors:** Ryan L Davis

**Affiliations:** 1 University of Sydney, Sydney, Sydney, Australia

*Mechanisms Associated with PINK1 Mutations in Parkinson's Disease*

 ABSTRACT

 Gene names should be in italics

 PINK1 and PTEN induced kinase 1 are the HGNC approved symbol and name – perhaps include the stable ID; HGNC:14581

 “Central to its pathogenesis is the PINK1 gene…” is a broad overgeneralisation that makes it seem like PINK1 is the only cause of PD and not more specific to monogenic early-onset familial PD.

 INTRODUCTION

 Parkinson’s disease is defined as (PD) in the first line, but the abbreviation is not consistently used thereafter

 There is a lot of information in the first paragraph that is not appropriately attributed to the original publications. Even a WHO report is mentioned but not referenced. Only the last sentence has an appropriate citation.

 “ *Within four years of diagnosis, there is a rapid and nearly complete loss of dopaminergic markers*

*in the dorsal striatum” *neglects the fact that there is a long pro-drome with progressive neurodegeneration. It also reflects the older onset progression of PD and not the early onset monogenic form that is relevant to PINK1. Might need to compare and contrast late vs early onset and idiopathic vs familial.

 Please check whether ‘variant’ is more appropriate than ‘mutation’

 Not sure how appropriate it is to reference a review for core research results. Reference the original citations where possible.

 Some citations are quite old. Suggest searching for more contemporary research evidencing the claims made.

 “PINK1 (PTEN-induced putative kinase 1) mutations represent the second most common cause of autosomal recessive early-onset Parkinson’s disease (EOPD), following *Parkin* mutations” suggests you are talking about the genes, and therefore PINK1 should be in italics and *Parkin* should be *PRKN*.

 2. DIVERSITY OF PINK1 MUTATIONS

 “Mutations in the PINK1 gene are strongly linked to PD” is suggestive that this is not definitive but rather remains controversial, which certainly isn’t the case. It would seem that this sentence is perfunctory.

 First paragraph has no references to support the information presented.

 Perhaps the second paragraph could be contextualised with the current extent of PINK1 variants and then perhaps that might help address the next comment.

 If the variants have been known for over 20 years how do they “…offers valuable guidance for the development of future therapeutic interventions.”?

 I would almost introduce normal PINK1 activity and physiology before talking about variants and how the dysfunction of the molecule underpins pathogenesis. As it stands, we’re learning about variants before really understanding the normal function and mechanisms of PINK1 activitiy.

 3. PINK1 AND THE PATHOGENESIS OF PD

 3.1 PINK1 MUTATIONS AND MITOCHONDRIAL PHOSPHORYLATION

 The section title suggests PINK1 phosphorylates mitochondria. Instead, a more accurate section title might be PINK1 mutations and target protein phosphorylation

 Figure legends should explain a figure as if it were standalone. Figure 1 only has a figure legend title and no supporting description of what is depicted. From author instructions for Reviews: “ **Titles and legends:** Each figure or table should have a concise title of no more than 15 words. A legend for each figure and table should also be provided that briefly describes the key points and explains any symbols and abbreviations used. The legend should be sufficiently detailed so that the figure or table can stand alone from the main text.” This is currently not the case for Figure 1.

 3.2 PINK1 MUTATIONS AND ABNORMAL MITOPHAGY PATHWAYS

 Parkin is introduced here as if it is the first time it is introduced with its function etc. yet it appears in all sections above

 While p62 was originally the best characterised autophagy receptor for Parkin-mediated mitophagy, there are several others that have been identified as being more prominent, including optineurin and NDP52. While NBR1 and TAX1BP1 are less prominent like p62.

 OVERALL

 Overall I found this short review to provide limited insight into the role of PINK1 in Parkinson’s disease with often outdated or incomplete supporting literature. PINK1 and Parkin have considerable influence outside of mitophagy, for instance PINK1 and Parkin were shown to be central to innate immunity in a knockout model where PD was induced by intestinal infection.

 Additionally, mitochondrial dysfunction can be the cause or corollary in PD and while this review takes the simplistic and repeated view of PINK1 and Parkin centrality to mitophagy, it is a limited view and negates the peripheral complexities and multifaceted nature of PD pathogenesis.

 As such, this review does nothing more than to reiterate information that have been presented ad nauseum without opinion or insight into the potential for therapeutic treatment that has thus far failed and remains an endeavour for the entire field.

 While the review is generally well written it lacks originality in insight and opinion that would drive the field in a particular direction, as a good review should do.

 As a result, I think this review is severely lacking and adds little to the literature that hasn’t already been presented numerous times previously.

Is the review written in accessible language?

Yes

Are all factual statements correct and adequately supported by citations?

Partly

Are the conclusions drawn appropriate in the context of the current research literature?

No

Is the topic of the review discussed comprehensively in the context of the current literature?

No

Reviewer Expertise:

Molecular Pathology, neurogenomics, Parkinson's Disease, Mitochondrial Disease

I confirm that I have read this submission and believe that I have an appropriate level of expertise to state that I do not consider it to be of an acceptable scientific standard, for reasons outlined above.

# Response

**Published:** 2026-03-15

**Authors:** Zheng Liu

**Affiliations:** Guilin Medical University, China

Mechanisms Associated with PINK1 Mutations in Parkinson's Disease

 ABSTRACT

 Gene names should be in italics

 PINK1 and PTEN induced kinase 1 are the HGNC approved symbol and name – perhaps include the stable ID; HGNC:14581

**Respond:** In accordance with the reviewer's suggestion, we have italicized all gene names (e.g., *PINK1*) throughout the manuscript to distinguish them from their protein products. Additionally, we have included the HGNC approved symbol, full name, and the stable ID (HGNC:14581) at the first mention of the gene in the Introduction.

 “Central to its pathogenesis is the *PINK1* gene…” is a broad overgeneralisation that makes it seem like *PINK1* is the only cause of PD and not more specific to monogenic early-onset familial PD.

**Respond:** We agree with the reviewer that the original statement was too broad. We have revised the text, “While most cases are sporadic, loss-of-function variants in the PINK1 gene are a primary cause of autosomal recessive early-onset PD.”. We have also clarified that studying *PINK1 *provides a critical window into mitochondrial dysfunction, which is a shared feature in both familial and sporadic PD.

 INTRODUCTION

 Parkinson’s disease is defined as (PD) in the first line, but the abbreviation is not consistently used thereafter

 Respond: We appreciate the reviewer's attention to detail. We have corrected this throughout the manuscript. Following the initial definition in the first sentence of the Introduction, the abbreviation "PD" is now used consistently in place of the full term.

 There is a lot of information in the first paragraph that is not appropriately attributed to the original publications. Even a WHO report is mentioned but not referenced. Only the last sentence has an appropriate citation.

**Respond: **We apologize for the insufficient citations in the original draft. We have now added the appropriate references for the epidemiological data and specifically cited the World Health Organization (WHO) report on Parkinson’s disease as requested.  

 “Within four years of diagnosis, there is a rapid and nearly complete loss of dopaminergic markers

 in the dorsal striatum” neglects the fact that there is a long pro-drome with progressive neurodegeneration. It also reflects the older onset progression of PD and not the early onset monogenic form that is relevant to PINK1. Might need to compare and contrast late vs early onset and idiopathic vs familial.

**Respond: **We appreciate this insightful comment. In the revised Introduction (Paragraph 2), we have addressed this by:

 1. Acknowledging the prolonged prodromal phase that precedes motor symptoms.

 2. Explicitly distinguishing the clinical phenotype of *PINK1*-linked PD from idiopathic cases. We now clarify that *PINK1* variants are associated with early-onset Parkinsonism (often before age 45) and a sustained response to levodopa, contrasting this with the older-onset progression of sporadic PD as suggested.

 Please check whether ‘variant’ is more appropriate than ‘mutation’

 Respond: We completely agree with the reviewer. In accordance with current genetic nomenclature guidelines, we have globally replaced the term "mutation" with "variant" throughout the manuscript to describe pathogenic genetic alterations.

 Not sure how appropriate it is to reference a review for core research results. Reference the original citations where possible. Some citations are quite old. Suggest searching for more contemporary research evidencing the claims made.

**Respond:** We appreciate this valid critique. We have comprehensively audited our reference list. We have removed over-reliance on older review articles and replaced them with highly relevant, contemporary primary research articles.

 “PINK1 (PTEN-induced putative kinase 1) mutations represent the second most common cause of autosomal recessive early-onset Parkinson’s disease (EOPD), following Parkin mutations” suggests you are talking about the genes, and therefore PINK1 should be in italics and Parkin should be PRKN.

**Respond:** PINK1 has been in italics and Parkin has been changed to PRKN.

 2. DIVERSITY OF PINK1 MUTATIONS

 “Mutations in the PINK1 gene are strongly linked to PD” is suggestive that this is not definitive but rather remains controversial, which certainly isn’t the case. It would seem that this sentence is perfunctory.

**Respond:** the sentence has been rewrote.

 First paragraph has no references to support the information presented.

**Respond:** the references has been added to the first paragraph.

 Perhaps the second paragraph could be contextualised with the current extent of PINK1 variants and then perhaps that might help address the next comment.

**Respond:** Thank you for the suggestion. I agree that providing a broader context on the current landscape of PINK1 variants—such as the distinction between pathogenic mutations and benign variants, or the known mutation hotspots—would strengthen the paragraph. This additional detail would not only ground the discussion but also serve as a natural bridge to the subsequent point in the comment."

 If the variants have been known for over 20 years how do they “…offers valuable guidance for the development of future therapeutic interventions.”?

**Respond:** We have extensively revised this section (now Section 3) to address the reviewer's concerns regarding clarity, referencing, and logical flow:

 1. Definitive Language: We have removed the tentative phrasing and now explicitly state that pathogenic variants cause a loss of function, disrupting the neuroprotective response.

 2. References Added: We have added the necessary citations to the opening paragraph to support the historical identification and structural clustering of these variants [Valente et al., 2004; Scholten et al., 2020].

 3. Updated Context: We have updated the text to reflect the current landscape, noting that over 70 variants have been cataloged, and we have categorized them by mechanism (kinase inactivation vs. protein instability).

 4. Removed Outdated Conclusion: We have removed the generic statement about "future therapeutic guidance." Instead, the section now concludes by summarizing the specific pathological outcome of these variants: the failure to sense mitochondrial depolarization and initiate mitophagy.

 I would almost introduce normal PINK1 activity and physiology before talking about variants and how the dysfunction of the molecule underpins pathogenesis. As it stands, we’re learning about variants before really understanding the normal function and mechanisms of PINK1 activity.

**Respond:** We are grateful for this excellent suggestion. We agree that a solid understanding of the physiological function of PINK1 is a prerequisite for discussing its pathogenicity. In the revised manuscript, we have inserted a new section entitled "Section 2. Physiological Role and Regulation of PINK1" immediately following the Introduction. In this section, we now detail:

 1. The canonical import of PINK1 into healthy mitochondria via the TOM/TIM complexes.

 2. Its proteolytic processing by MPP and PARL.

 3. Its rapid degradation via the N-end rule pathway.

 4. Its function as a molecular sensor for mitochondrial membrane potential. This new section provides the necessary biological context before we introduce the pathogenic variants in the subsequent section (now Section 3).3. PINK1 AND THE PATHOGENESIS OF PD

 3.1 PINK1 MUTATIONS AND MITOCHONDRIAL PHOSPHORYLATION

 The section title suggests PINK1 phosphorylates mitochondria. Instead, a more accurate section title might be PINK1 mutations and target protein phosphorylation

**Respond: **We agree with the reviewer that the original title was imprecise. In the revised manuscript, we have renamed this section (now Section 4.1) to " *PINK1* Variants Impair Phosphorylation of Downstream Substrates." This title more accurately reflects the mechanistic reality that PINK1 targets specific proteins—namely Parkin and Ubiquitin—rather than the mitochondrial organelle as a whole.

 Figure legends should explain a figure as if it were standalone. Figure 1 only has a figure legend title and no supporting description of what is depicted. From author instructions for Reviews: “Titles and legends: Each figure or table should have a concise title of no more than 15 words. A legend for each figure and table should also be provided that briefly describes the key points and explains any symbols and abbreviations used. The legend should be sufficiently detailed so that the figure or table can stand alone from the main text.” This is currently not the case for Figure 1.

**Respond:** We apologize for this omission. We have provided a comprehensive, standalone legend for Figure 1 that thoroughly explains the depicted mechanisms, including basal turnover, mitophagy initiation (highlighting OPTN/NDP52), and the disease state. All abbreviations used in the figure are now explicitly defined in the legend. Additionally, we have ensured that Figure 1 is properly cited and integrated within the relevant mechanistic sections of the main text to guide the reader effectively.

 3.2 PINK1 MUTATIONS AND ABNORMAL MITOPHAGY PATHWAYS

 Parkin is introduced here as if it is the first time it is introduced with its function etc. yet it appears in all sections above

 While p62 was originally the best characterised autophagy receptor for Parkin-mediated mitophagy, there are several others that have been identified as being more prominent, including optineurin and NDP52. While NBR1 and TAX1BP1 are less prominent like p62.

**Respond:** We are grateful to the reviewer for their careful reading and for pointing out this critical evolution in the field. We have made two major revisions to this section (now Section 4.2) to fully address these concerns:

 1. Removal of redundant Parkin introduction: We apologize for the redundancy. With the addition of the new "Section 2. Physiological Role and Regulation of PINK1" earlier in the manuscript, Parkin's baseline function is now appropriately introduced upon its first mention. We have carefully revised Section 4.2 to remove the repetitive introduction of Parkin, ensuring a smoother logical flow.

 2. Updating the mitophagy receptor consensus: We have significantly revised the text to reflect the current consensus on autophagy receptors. As the reviewer correctly pointed out, we now explicitly state that Optineurin (OPTN) and NDP52 are the primary receptors essential for mitophagy. We also briefly acknowledge that p62, NBR1, and TAX1BP1 play less prominent roles. Furthermore, we now detail how PINK1-mediated phosphorylation of ubiquitin serves as the specific signal to recruit OPTN and NDP52. Key citations have been added to support this updated, contemporary perspective.OVERALL

 Overall I found this short review to provide limited insight into the role of PINK1 in Parkinson’s disease with often outdated or incomplete supporting literature. PINK1 and Parkin have considerable influence outside of mitophagy, for instance PINK1 and Parkin were shown to be central to innate immunity in a knockout model where PD was induced by intestinal infection.

 Additionally, mitochondrial dysfunction can be the cause or corollary in PD and while this review takes the simplistic and repeated view of PINK1 and Parkin centrality to mitophagy, it is a limited view and negates the peripheral complexities and multifaceted nature of PD pathogenesis.

 As such, this review does nothing more than to reiterate information that have been presented ad nauseum without opinion or insight into the potential for therapeutic treatment that has thus far failed and remains an endeavour for the entire field.

 While the review is generally well written it lacks originality in insight and opinion that would drive the field in a particular direction, as a good review should do.

 As a result, I think this review is severely lacking and adds little to the literature that hasn’t already been presented numerous times previously.

**Respond: **We are deeply grateful for this profound and constructive critique, which has fundamentally elevated the quality of our manuscript. We fully agree that our previous draft was too narrowly focused on canonical mitophagy and lacked forward-looking translational insights. To address this, we have made substantial additions:

 1. Expansion Beyond Mitophagy (Innate Immunity & Gut-Brain Axis): We have added a completely new section (Section 4.3: Beyond Mitophagy: PINK1 and Neuroinflammation). As astutely suggested by the reviewer, we now extensively discuss the role of PINK1 as a critical immunological checkpoint. We specifically highlight the seminal Nature study demonstrating that intestinal infection triggers autoimmune mechanisms in *Pink1*-knockout mice via mitochondrial antigen presentation (Matheoud et al., 2019) , as well as the activation of the cGAS-STING pathway.

 2. Translational Relevance and Therapeutic Insight: We have added another new section (Section 4.4: Therapeutic Implications) to provide the missing "insight and opinion" on drug development. We critically evaluate novel precision medicine strategies, specifically highlighting the amplification of kinase activity (via Kinetin) and the promising strategy of bypassing mitophagy blockades using USP30 inhibitors.

 3. We believe these major revisions transform the manuscript from a mere descriptive summary into a critical, forward-looking review that offers a new framework for understanding PINK1 pathogenesis and developing targeted therapies.

# Reviewer report: Reviewer response for version 1

**DOI:** 10.5256/f1000research.187509.r440954

**Published:** 2025-12-29

**Authors:** Eman Ezzeldien Mohamed

**Affiliations:** 1 Beni-Suef University, Beni-Suef, Egypt

Reviewer:

 This manuscript addresses an important topic by reviewing the mechanisms linking PINK1 mutations to Parkinson’s disease. The review is generally well-referenced and provides informative coverage of mitochondrial dysfunction, autophagy, and oxidative stress.

 1. The Introduction should more clearly define the scope of the review and highlight existing knowledge gaps. Several sections remain largely descriptive, and greater critical analysis of the literature is needed.

 2. the Review  should better explain the mechanistic reasons behind the reported pathological outcomes.

 3.  the clinical and translational relevance of PINK1 mutations could be more explicitly addressed. Improving integration between the figure and the main text would also enhance clarity.

Is the review written in accessible language?

Yes

Are all factual statements correct and adequately supported by citations?

Partly

Are the conclusions drawn appropriate in the context of the current research literature?

Yes

Is the topic of the review discussed comprehensively in the context of the current literature?

Yes

Reviewer Expertise:

physiology

I confirm that I have read this submission and believe that I have an appropriate level of expertise to confirm that it is of an acceptable scientific standard.

# Response

**Published:** 2026-03-15

**Authors:** Zheng Liu

**Affiliations:** Guilin Medical University, China

This manuscript addresses an important topic by reviewing the mechanisms linking PINK1 mutations to Parkinson’s disease. The review is generally well-referenced and provides informative coverage of mitochondrial dysfunction, autophagy, and oxidative stress.

 1. The Introduction should more clearly define the scope of the review and highlight existing knowledge gaps. Several sections remain largely descriptive, and greater critical analysis of the literature is needed.

 Respond: We agree. We revised the Introduction's final paragraph to clearly define the review's scope and highlight knowledge gaps, specifically PINK1's non-mitophagic roles in innate immunity. We also cited seminal studies on the gut-brain axis and inflammation, such as Matheoud et al. (2019), to enhance our critical analysis.

 2. the Review should better explain the mechanistic reasons behind the reported pathological outcomes.

 Respond: Thank you for the suggestion. We significantly deepened the mechanistic discussions. In Section 3, we detail how specific variants fail to phosphorylate Ubiquitin/Parkin at Ser65. In Section 4.2, we explain how this failure blocks the recruitment of key autophagy receptors (OPTN and NDP52), leading to the accumulation of ROS-producing mitochondria that mechanically drives neurodegeneration.

 3. the clinical and translational relevance of PINK1 mutations could be more explicitly addressed. Improving integration between the figure and the main text would also enhance clarity.

 Respond: We appreciate these suggestions and made two major updates:

 1.Translational Relevance: We added a new section (Section 4.4: Therapeutic Implications) exploring precision medicine strategies, highlighting neo-substrates (e.g., Kinetin) and USP30 inhibitors.

 2. Figure Integration: We rewrote the legend for Figure 1 so it fully explains the mechanisms and stands alone. We also improved its integration by citing it at key mechanistic junctures (e.g., Sections 2 and 4.2) in the main text.
